# Mobile-Social Learning for Continuing Professional Development in Low- and Middle-Income Countries: Integrative Review

**DOI:** 10.2196/32614

**Published:** 2022-06-07

**Authors:** Dominique Guillaume, Erica Troncoso, Brenice Duroseau, Julia Bluestone, Judith Fullerton

**Affiliations:** 1 Jhpiego Baltimore, MD United States; 2 School of Nursing Johns Hopkins University Baltimore, MD United States

**Keywords:** digital learning, continuing medical education, mHealth, peer learning, mentorship, health systems, global health, mobile phone

## Abstract

**Background:**

Access to continuing professional development (CPD) for health care workers in low- and middle-income countries (LMICs) is severely limited. Digital technology serves as a promising platform for supporting CPD for health care workers by providing educational content virtually and enabling virtual peer-to-peer and mentor interaction for enhanced learning. Digital strategies for CPD that foster virtual interaction can increase workforce retention and bolster the health workforce in LMICs.

**Objective:**

The objective of this integrative review was to evaluate the evidence on which digital platforms were used to provide CPD to health care workers and clinical students in LMICs, which was complemented with virtual peer-to-peer or mentor interaction. We phrased this intersection of virtual learning and virtual interaction as *mobile-social learning*.

**Methods:**

A comprehensive database and gray literature search was conducted to identify qualitative, quantitative, and mixed methods studies, along with empirical evidence, that used digital technology to provide CPD and virtual interaction with peers or mentors. The PRISMA (Preferred Reporting Items for Systematic Reviews and Meta-Analyses) guidelines were followed. Eligible articles were written in English, conducted in an LMIC, and used a mobile device to provide CPD and facilitate virtual peer-to-peer or mentor interaction. Titles, abstracts, and full texts were screened, followed by an assessment of the quality of evidence and an appraisal of the articles. A content analysis was then used to deductively code the data into emerging themes.

**Results:**

A total of 750 articles were identified, and 31 (4.1%) were included in the review. SMS text messaging and mobile instant messaging were the most common methods used to provide continuing education and virtual interaction between peers and mentors (25/31, 81%). Across the included articles, participants had high acceptability for using digital platforms for learning and interaction. Virtual peer interaction and mentorship were found to contribute to positive learning outcomes in most studies (27/31, 87%) through increased knowledge sharing, knowledge gains, improved clinical skills, and improved service delivery. Peer-to-peer and mentor interaction were found to improve social support and reduce feelings of isolation (9/31, 29%). There were several challenges in the implementation and use of digital technology for mobile-social learning, including limited access to resources (eg, internet coverage and stable electricity), flexibility in scheduling to participate in CPD, and sociobehavioral challenges among students.

**Conclusions:**

The summary suggests that mobile-social learning is a useful modality for curriculum dissemination and skill training and that the interface of mobile and social learning serves as a catalyst for improved learning outcomes coupled with increased social capital.

## Introduction

### Background

The shortage of health care providers in low- and middle-income countries (LMICs) places an insurmountable strain on health care systems. The World Health Organization estimates that nearly 57 countries lack an approximate 4.2 million health care providers (physicians, nurses, midwives, and allied health professionals) [1,2]. The strengthening of health care systems in LMICs requires multifaceted approaches to the training and retention of health care workers to meet clinical needs [3]. Continuing professional development (CPD) for health care providers is essential for the development and application of health care practices and policies necessary for health promotion, disease prevention and management, and fostering sustainable health systems. Although many high-income countries require health care workers to participate in regular CPD, many LMICs do not have such regulations or policies. Traditionally, in-person CPD training has been the primary method of providing health care education for health care providers in LMICs [4]. However, health care workers in low-resource settings, especially those in rural areas, face substantial logistical barriers to accessing in-person CPD programs (eg, cost of travel and inflexibility in scheduling). Thus, access to such programs is remarkably limited, especially when there is a lack of provider engagement [4]. Research has demonstrated that health care workers in LMICs are more likely to have higher motivation, satisfaction, and retention when they are provided with access to continuing education [3,5-7]. In countries with limited resources, addressing health care worker shortages and service needs requires tailored, cost-effective approaches for training, supervising, and mentoring health care workers. It is imperative for such approaches to minimize strain on already burdened health care systems while simultaneously providing instructional experiences that trainees need to successfully perform their jobs [8].

### Mobile-Social Learning to Support CPD

Digital education strategies have gained momentum over the last decade in low-resource settings for the provision of CPD to health care workers. Digital education encompasses various modalities of learning, including but not limited to offline and web-based computer-based education, gamification, massive open web-based courses, virtual reality environments, augmented reality, virtual patient simulations, and mobile digital education [9]. Given the expansion of mobile phone use in LMICs, leveraging digital health strategies through the use of mobile technology has the potential to alleviate significant health system challenges [10,11]. CPD that is provided through mobile devices can support workplace-based practical training, reduce in-person instruction time, support social peer learning, and allow programs to reach a greater number of providers, especially those practicing in remote locations [4]. Virtual training is affordable and can be easily adapted as new information is discovered to update providers with the latest information and clinical developments. Furthermore, virtual CPD that is provided through mobile technology has demonstrated high feasibility and acceptability among health care providers in LMIC settings [4,12-14]. Mobile platforms can be used to not only provide CPD education but also complement CPD through peer-to-peer and mentorship engagement and interaction. Although there have been a number of studies that have evaluated the efficacy of digital and mobile health (mHealth) technology for providing CPD to health care workers, few studies have evaluated the potential of digital health, specifically mHealth, in providing peer interaction and mentorship for sustained education and training. The investigation of mobile-networked communication technology for health care provider CPD has been relatively understudied in low-income settings [15].

Jhpiego (a Johns Hopkins University affiliate) has supported CPD for health care providers and capacity building in multiple low-resource settings for nearly 50 years. In leveraging strategies to increase the accessibility of CPD programs, a heightened focus has been placed on using digital training approaches, including the provision of CPD through mobile phones [16-18]. Jhpiego recognizes that delivering lean, just-in-time learning via mobile devices can support workplace-based practical training, reduce in-person instruction time, support social peer learning, and allow programs to reach greater numbers of providers. As access to mobile technology continues to increase among health care providers in LMICs, so does access to platforms that foster virtual interaction and communication, such as mobile instant messaging services (eg, WhatsApp) and social media (eg, Facebook, Instagram, and Twitter). We believe that health workers should receive greater social support to improve retention rates, improve morale, and accelerate the potential of social learning. Such findings have been demonstrated in high-income countries, where social support and virtual interaction were found to foster understanding and learning among health care professionals and clinical students [19,20]. Social and mobile platforms can be used for learning and support broader, facility-based quality improvement efforts with scalable efficiency. For these purposes, we have combined the terms into a single phrase, *mobile-social*, to describe this key intersection that warrants further investment for achieving greater capacity-building impact. We are defining mobile-social learning as a new methodology that is powerful for supporting health care providers to improve their clinical capacity, learning, and performance. A mobile-social learning approach incorporates the following two aspects: the use of mobile technology to increase access to digital learning opportunities and social platforms that encourage the social aspect of learning by facilitating professional networks for the sharing of experiences and exchange of knowledge through virtual communication.

Through mobile-social learning, mobile distance education is provided to users and supplemented by web-based real-time discussions and a collaborative learning approach. This allows for opportunities for students to collaborate to construct knowledge while promoting the development of learning communities and supporting the learning process [21]. As part of Jhpiego’s commitment to make learning available and convenient to health workers anytime, anywhere, whether in the workplace, on the road, or at home, we see fostering this modality for learning and supporting peer learning as a great investment. Given the dearth of literature that has assessed the efficacy and outcomes of mobile-social learning in LMICs, the purpose of this integrative review was to explore the potential of mobile-social learning to support capacity building and improve the quality of CPD for health care providers in LMICs.

## Methods

### Search Strategy

A comprehensive literature search was undertaken using the PRISMA (Preferred Reporting Items for Systematic Reviews and Meta-Analyses) recommendations to guide the search and review process. Peer-reviewed literature published between January 2016 and March 2021 was searched for on the PubMed, CINAHL, and Embase bibliographic databases. A gray literature search was also undertaken using Digital Square and the US Agency for International Development mHealth database. Hand searches of references from articles that were populated from the search were also conducted to identify relevant articles that may not have been identified using the search strategy. Searches were conducted separately on each database using controlled vocabulary supplemented with keywords and Medical Subject Headings terms combined with the Boolean operators *OR* and *AND*. The key terms and medical subject heading terms included concepts pertaining to continuing education, virtual training, health care providers, e-learning, mentorship, peer-to-peer interaction, and LMICs (Multimedia Appendix 1).

### Eligibility Criteria

Studies were included if they were written in English, took place in an LMIC, and used mobile devices (eg, cellular phones, smartphones, tablets, palmtops, and pocket PCs) to provide continuing education and skill training to health care providers or preprofessional students. We included studies in which mobile platforms that fostered the concept of mobile-social learning were used either solely or in conjunction with a traditional face-to-face learning approach (eg, blended learning approach) [9]. Interventions using mobile-social learning were defined as any teaching, learning, or training intervention along with virtual interaction delivered using wireless networking, mobile telecommunication technology, multimedia messaging services, or SMS text messaging through a mobile device [9]. Virtual interaction among the eligible articles had to consist of either peer-to-peer interactions or mentor interactions that bolstered learning and professional support. Articles that did not evaluate student learning outcomes from virtual continuing education programs and that solely focused on program design and feasibility were excluded.

Peer-reviewed studies were included in our synthesis in addition to gray literature that was not peer-reviewed. Gray literature included relevant programmatic reports, case reports, research reports, presentations, and issue papers published by government entities, nongovernmental organizations, and private organizations. We included non–peer-reviewed gray literature given the possibility of limited published research on this topic coupled with the recent shift toward digital learning modalities concurrent with the COVID-19 pandemic. Thus, including gray literature allowed for an opportunity to highlight relevant work that may not have otherwise been identified while providing a balanced view of the evidence given the lack of peer-reviewed studies on this topic [22]. Furthermore, this method mitigated the risk of publication bias, which could potentially limit the availability of research in a field that is novel and rapidly growing [11,22].

### Study Identification and Selection

The search yielded a total of 750 articles (PubMed: n=484, 64.5%; CINAHL: n=202, 26.9%; Embase: n=31, 4.1%; Digital Square: n=22, 2.9%; US Agency for International Development mHealth database: n=10, 1.3%) in addition to 2 articles that were identified from a hand search of previously published systematic reviews that assessed the use of digital health for educating health care providers. Articles were imported into Mendeley (Elsevier) and subsequently uploaded to the systematic review tool *Rayyan QCRI* (Rayyan Systems Inc). Duplicates were identified and excluded (104/750, 13.9%), thus resulting in a total of 646 articles that were screened. Authors DG, JB, and ET conducted title and abstract screening and full-text screening. After title and abstract screening of the 646 articles, a total of 602 (93.2%) were excluded, with 44 (6.8%) remaining for full-text screening. Disagreements among the articles were identified and resolved through consensus (DG, ET, and JB). Articles were then graded on their quality of evidence using the Johns Hopkins Nursing Evidence Level and Quality Guide [23]. This tool was selected as it has been extensively used in the literature and provides grading criteria for peer-reviewed studies along with experiential, nonresearch evidence (eg, case reports, quality improvement guidelines, and programmatic reports). The following factors were assessed in evaluating the studies and determining the quality of evidence: generalizability of the results, sample size, control, consistency of the results, methodology, limitations, conclusions, and recommendations [23]. After the articles were evaluated, the research team made final agreements for inclusion.

### Data Extraction and Analysis

Once the articles were agreed upon for inclusion by the research team (DG, JB, and ET), data from the included articles were extracted (DG). The extracted data from the included studies consisted of the following categories: the country in which the study was conducted, study design and methods, study aims, participant characteristics, clinical focus, intervention and comparisons, outcome measures, key findings, and limitations. An integrated approach using data conversion was used, in which quantitative and qualitative data were used to address the research question. Quantitative data were transformed into qualitative themes as described in Tashakkori and Teddlie [24]. The qualitization of quantitative data involves converting quantitative data into qualitative themes in which data are theoretically grouped based on concepts measured from cross-sectional survey data [25-28]. Quantitative data were operationalized based on the concepts that were measured, and clusters of numeric data were transformed into qualitative themes. Quantitative and qualitative data were inductively coded line-by-line followed by the codes being categorized into broader themes [28,29]. The identified themes included the following: student perspectives on mobile-social learning, forms of interaction and communication within mobile-social platforms, learning outcomes, and challenges to and facilitators of mobile-social learning.

### Conceptual Framework

**Figure 1:**
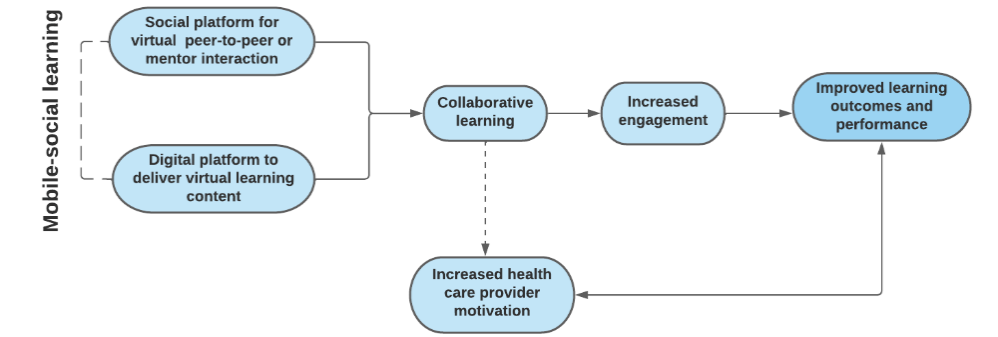
Mobile-social learning conceptual framework.

Findings from the synthesis of the included studies were used to develop a conceptual framework depicting mobile-social learning (Figure 1). In this conceptual framework, virtual learning and virtual engagement with peers or mentors foster collaborative learning. Collaborative learning results in increased engagement followed by improved learning outcomes and performance. Collaborative learning can also increase health care provider motivation and lead to improved outcomes and performance. This relationship can also be reciprocal to improved learning outcomes, contributing to increased motivation among health care providers. We anticipate that this model will be used in providing CPD for clinical professionals, community health workers, and preclinical and clinical students on a variety of platforms in low-resource settings where there may be significant challenges in implementing and accessing face-to-face CPD.

## Results

### Included Studies

**Figure 2:**
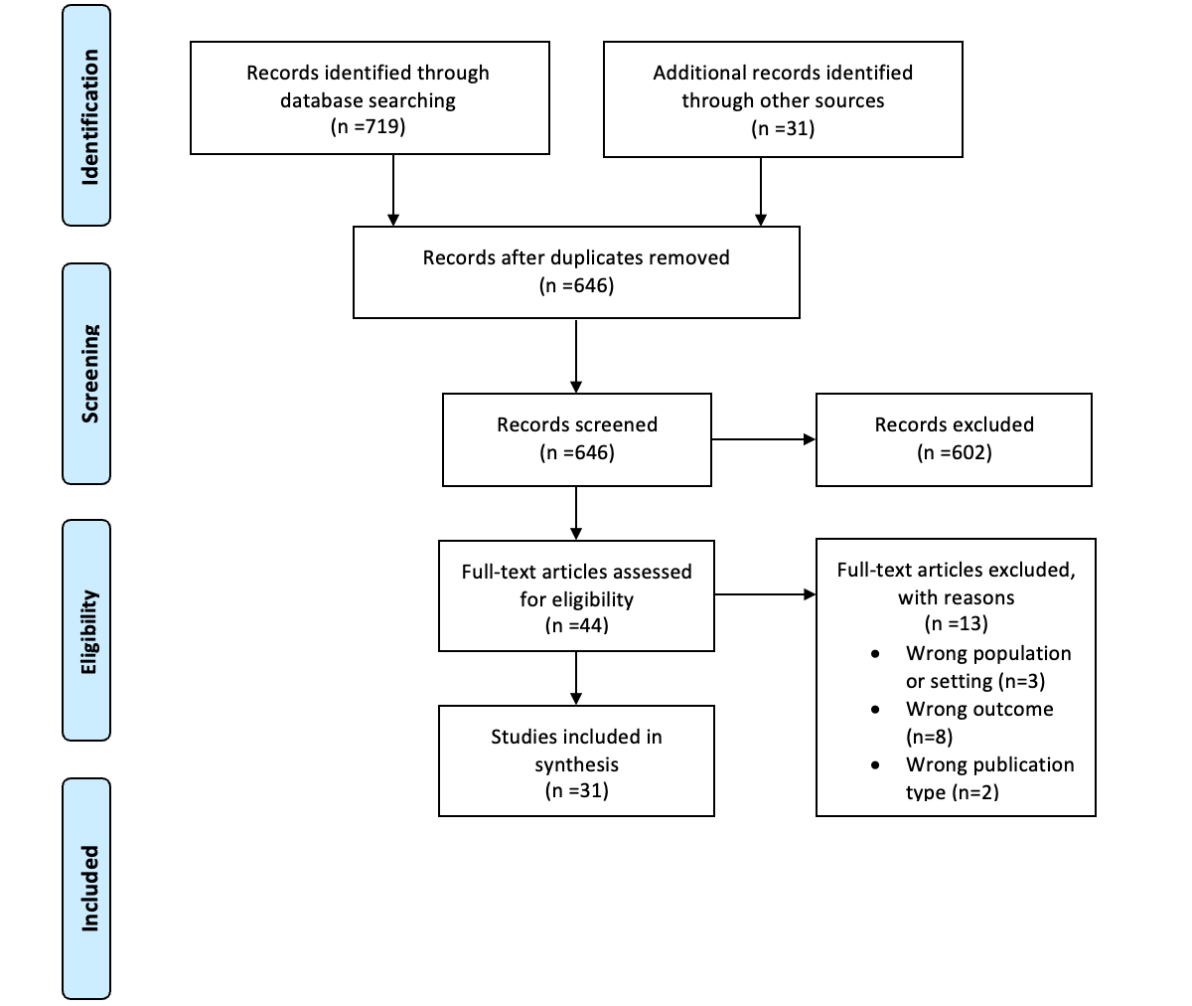
PRISMA (Preferred Reporting Items for Systematic Reviews and Meta-Analyses) flow diagram.

A total of 31 articles were selected for inclusion (Figure 2). Among the included articles, sub-Saharan Africa (28/31, 90%) was the most represented geographical region [1,8,15-17,30-52]. Countries in Southeast Asia were represented in 10% (3/31) of the studies [45,53,54], and 3% (1/31) of the studies included participants across Africa, Asia, and South America [1]. A wide array of health care cadres was targeted across the studies, including nurses, physicians, midwives, community health workers, public health specialists, hospital administrators, and health officers. In addition, several studies (7/31, 23%) included preprofessional clinical trainees (eg, nursing and medical students) [32-34,37,50-52]. Of the 31 studies, 9 (29%) were qualitative in design and were conducted using descriptive thematic analysis, in-depth interviews, or focus groups [8,15,30,32,34,36,40,41,50]. A total of 39% (12/31) of the studies [1,16,31,33,35,37,45-48,51,54] were quantitative, with most quantitative studies (10/12, 83%) having observational cross-sectional designs. Of the 31 studies, 10 (32%) used both quantitative and qualitative data collection [17,38,39,42-44, 49,52,53,55] (Multimedia Appendix 2 [1,8,15-17,29-54]).

There was diversity in the clinical topics that were represented among the continuing education interventions. Nearly half (14/31, 45%) of the interventions focused on clinical topics pertaining to sexual and reproductive health (ie, maternal health, basic emergency obstetric and newborn care, HIV and AIDS prevention and treatment, treatment of sexually transmitted infections, cervical cancer screening, and family planning) [1,8,16,17,30,31,40-42,47,48,52,54,55]. Additional clinical topics included primary care, general nursing practice and skills, research, malnutrition, anesthesia, pediatric hematology-oncology, integrated management of childhood illnesses, nephrology, and orthopedics (Multimedia Appendix 3 [1,8,15-17,29-54]).

### Interventions

Most articles (25/31, 81%) used texting, mobile instant messaging, or SMS text messaging to provide continuing education content either as a stand-alone intervention or in addition to other digital learning tools and blended learning formats (eg, coupling SMS text messaging with traditional face-to-face learning) [8,15-17,30-38,40,41,43,45,46, 48-52,55,56]. WhatsApp was the most commonly used platform among the studies that used messaging services (17/31, 55%) [8,15,30-34,36,37,43,46,48,50-52,55,56]. Telephone calls were included in 16% (5/31) of the studies and were primarily used between students and mentors to discuss and reinforce learning content and for students to obtain feedback from mentors [16,41,45,46,53]. Web-based courses that facilitated web-based interactive discussions and commentary were used in 19% (6/31) of the studies [1,42-46]. Of these 6 studies, 3 (50%) coupled web-based courses with social media platforms such as Facebook, Twitter, and Google groups, which served as an adjunct to the course in facilitating learning and peer interaction and engagement [1,43,44]. A total of 3% (1/31) of the studies used Facebook as the sole platform in both providing educational content and facilitating discussions between peers and mentors [39] (Multimedia Appendix 3).

### Acceptability

Across the studies that assessed student acceptability and student satisfaction with the mobile-social interventions (16/31, 52%), most participants strongly endorsed using mobile-social learning for continuing education and were highly satisfied with the platform [1,8,15,32,36-42,46,49,51,52,54,55]. Students regarded the learning platforms as easily accessible, informative, and user-friendly, with the social components improving communication and knowledge sharing and fostering real-time feedback. Students in a number of studies (12/16, 75%) specifically highlighted the ability to receive real-time feedback from peers or mentors as beneficial to clinical practice, knowledge gains, and team building [8,15,31, 34-37,39,40,50,52,53].

### Student Engagement

Articles that measured student engagement (16/31, 52%) within the mobile-social learning platforms reported high levels of use among students [1,15,31,33,36-39,43-45,48,50-52,55]. However, the association between levels of engagement and learning outcomes varied among the studies. Woods et al [31] noted that students who regularly followed the WhatsApp learning group had a clinically significant increase in the odds of having higher confidence in managing their patients (odds ratio 8.44, 95% CI 2.33-35.23; *P*<.05). Abiodun et al [37] noted that, although students were highly engaged within WhatsApp groups, there were no significant associations between the frequency of reading messages and social-professional outcome measures. Among the studies that coupled web-based learning modules with social media interaction and discussions through Facebook and WhatsApp, module completion rates were significantly higher compared with standard massive open web-based course completion [1,30,43,44].

### Interaction With Peers

High levels of peer engagement and interaction were reported among the included studies (19/31, 61%) [1,15,30,32,34,38-40,42-44,46,49-52,54,57]. A dominant theme that arose among the studies that assessed peer interaction was the ability to engage in active knowledge sharing. Interventions that fostered peer engagement and interaction through messaging and discussion forums found that participants viewed the interactive component and commentary as beneficial [1,15,30,32,34,38-40,42-44,46,49-52,54,57]. CPD programs that were augmented with social media found that peer support was facilitated through social media platforms, which allowed for real-time interactions and peer feedback and greater understanding of course material [1,30,43,44]. The inclusion of a peer support network was suggested by students in a study that solely focused on virtual mentorship for cervical cancer screening among nurses and did not include a virtual peer-to-peer interaction component [40]. Students in this study stated that, although they were actively engaged with mentors virtually, they strongly believed that including a peer support network or chat room to actively discuss clinical cases and medical imaging in real time would be beneficial [40].

### Interaction With Mentors

Mentorship occurred at varying degrees (eg, pairing a student with a master mentor, assigning groups of students to a mentor, and facilitators providing feedback to students in discussion groups). Among the studies that included virtual mentorship (24/31, 77%), students reported positive interactions with mentors [1,8,15-17,34,35,38-41,43-54]. Mentorship occurred through phone calls, texting, and video calls in which mentors were able to provide remote support, real-time feedback, and guidance to students along with reinforcement of key learning messages and skills gained from educational modules and training sessions. In studies where a blended training approach was used, students emphasized the importance of having virtual access to an expert for further questions and case discussions after the initial on-site training [40,45]. Asiedu et al [37] noted that, despite receiving positive feedback on the mobile mentoring component from students, several students and mentors voiced concerns that mobile mentoring on its own was insufficient for posttraining follow-up and support. A few mentors noted that it was difficult to sustain the process of repeated telephone calls to students and that certain students were not being honest about progress [37]. The intervention implemented by Feldacker et al [42] did not contain a virtual mentorship component; however, students highly suggested the inclusion of virtual mentoring opportunities to supplement learning. Similarly, an intervention in Kenya that used WhatsApp mobile messaging for family planning learning did not include a mentorship component; however, students voiced the need for more mentorship support [55].

### Learning Outcomes

Students who used mobile-social platforms reported positive learning outcomes in most of the included articles (27/31, 87%) [1,8,15,17,30-39,41-48,50,52,54,55,58]. Learning outcomes included knowledge gains, improved clinical skills, positive influences on clinical practice, and improved quality of service delivery. Students emphasized the educational benefits of having live case discussions with peers or mentors via the mobile-social learning platforms, in which patient-related questions could be addressed in real time along with consulting with peers or mentors about complex clinical cases [8,15, 30,36,38,46,50,52,54]. Across the studies that combined face-to-face learning with mobile-social learning (8/31, 26%), digital platforms that supported mobile-social learning were found to be instrumental in bridging the periods between face-to-face meetings through the ability of the participants to engage in continuous communication and feedback [52]. In 13% (4/31) of the studies where mobile messaging through peer groups was used, participants stated that previous case discussions with peers were used as a resource to which they referred when presented with a complicated clinical case in their practice. In addition, students cited using old case discussions saved in group chats for self-study [31,34,38,41].

Of the 31 studies, 3 (10%) [16,17,49] compared mobile-social learning platforms with traditional face-to-face interventions, but these studies yielded varied results regarding learning outcomes. Yigzaw et al [16] and Muhe et al [49] found no significant differences between groups in the gains in knowledge scores, whereas Ugwa et al [17] reported that the virtual learning arm, which included mobile mentoring, led to better skill performance at all assessment points compared with the traditional arm, with the virtual arm performing better in all competencies at 3 and 12 months after training.

### Social Capital and Professional Integration

Social capital characterizes the relationships and interactions between members of a social group [59]. Social capital encompasses a culture of trust and tolerance in which extensive networks of voluntary associations emerge that facilitate coordination and cooperation for mutual benefit [60,61]. In medicine, social capital has been tied to the realization of lifelong learning opportunities, with digital engagement contributing to the development and maintenance of social capital [51,52]. Social capital typologies among the included articles included emotional support, the formation of deeper social connections with peers and colleagues, networking, and non–work-related communication [8,15,30,33,37,39,42,51,52]. Students in the studies that measured social capital (9/31, 29%) cited that virtual interaction with peers or mentors reduced feelings of isolation. Participants stated that interactions helped them maintain existing relationships while also developing and strengthening new social ties, thus promoting professional integration and improving team dynamics [33,34,37,51]. Pimmer et al [33] and Abiodun et al [37] noted that participants with higher levels of active engagement also felt less isolated professionally and had higher levels of social capital.

### Challenges and Success Factors

Although positive feedback was elicited from students regarding mobile-social learning, there were several challenges in the implementation and use of the learning modality. Resource challenges related to power cuts and maintenance of uninterrupted internet access were primary barriers to the use of the mobile-social learning platforms in several studies (14/31, 45%) [8,15,31,32,38,40,42-46,50,52,54]. Ugwa et al [17] cited additional resource challenges pertaining to the unavailability of equipment for students to translate what they learned on the web into practice. The lack of time to participate in learning programs because of conflicts with clinical schedules was a challenge noted in 13% (4/31) of the studies [8,17,42,44]. However, 19% (6/31) of the studies highlighted that mobile-social programs allowed for increased flexibility of learning that could easily fit into students’ schedules and reduce the need for travel to access face-to-face continuing education programs, which could be challenging for health care providers located in rural areas [1,32,42,43,49,54]. The costs of data bundles and messaging services to support the programs were cited as barriers in 10% (3/31) of the studies [41,49,52]. However, a few studies (6/31, 19%), including those that mentioned cost as a barrier, stated that, over the long term, distance learning approaches were more cost-effective compared with traditional face-to-face programs [8,16,41,43,48,49]. Sociobehavioral challenges were also noted, such as certain users engaging in inappropriate non–work-related discussions on the learning platforms, low levels of active participation, and low digital literacy [15,38,45,50]. In addition, hesitancy among students to interact on platforms because of fear, embarrassment, or lack of knowledge or awareness was cited as a barrier [38,40]. Success factors that were noted among the interventions included tailor-made content to meet the needs of the participants, the flexibility of being able to access learning content anytime and anywhere, and features that promoted ongoing engagement and personal interactivity with peers and mentors [1,8,15,17,30,32-35,37-39,42-47,49,51,52,54]. Peer and mentor interaction allowed for direct personalized feedback, the application of educational content from theory to clinical practice by the students, self-direction for learning, and active learning. Interaction with peers and mentors heightened confidence and promoted empowerment among students, thus leading to increased motivation to maintain newly acquired knowledge and skills [31,40,41].

## Discussion

### Principal Findings

The use of a wide variety of mobile platforms has become a common adjunct to traditional classroom-based pedagogy and, in many cases, is the default strategy for curriculum dissemination in many countries. However, even in the era of increased uptake of mobile platforms for learning, multifaceted approaches are needed for the diversity of learning needs and preferences [42,62-64]. This integrative review demonstrates that mobile platforms that foster mobile-social learning can serve as an innovative method for providing health care workers and preprofessional students with skill training and education along with virtual interactive components. A major advantage of these strategies is the ability to reach a widely dispersed learner population residing in diverse geographic locations [15,30,40,54]. Access to learning can be independent of time specificity and open to the availability of repetitive exposure to content in support of mastery learning. This evidence synthesis demonstrates the added value of social learning networks as interventions that enhance the utility and effectiveness of internet-based learning platforms whether as independent dissemination strategies or as part of blended learning (hybrid) teaching and learning approaches. Social networks serve as the interface between teachers, mentors, independent learners, and the network of learners engaged in the exploration of any single topic [65,66]. Social networks through mobile platforms can be used to facilitate mobile-social learning as a means of deeper exploration of the understanding of content through peer engagement and interactive discussion and the giving and receiving of feedback [66,67]. In addition, this review highlighted the impact of mobile-social learning on reducing feelings of professional isolation and increasing social capital among health care workers. Increased social connectedness and social presence can play a key role in team building and increasing health care worker motivation and confidence [68]. Improved health care worker motivation may serve as a critical component in reducing attrition in LMICs, thus improving workforce capacity within health systems.

The studies included in this review offered evidence for the acceptability of the use of mobile platforms as an instructional modality, viewing them as accessible and user-friendly. Learners recognized and valued the addition of social interaction strategies via these mobile platforms as measures to receive affirmation of learning. This reinforcement offered by peers and mentors affected the confidence of students when translating learning into clinical practice. The interface of mobile learning with social engagement in the learning process offered the opportunity for meaningful interaction with a wider network of learners and health workers. The ability to engage in real-time discussion of clinical topics and resolution of clinical challenges diminished feelings of isolation, extended the professional network for collaboration when the next need might arise, and expanded the opportunity to remain current with emerging evidence underpinning clinical practice, thus affecting the quality of service delivery.

The evidence synthesis also highlighted the challenges that are inherent in the use of mobile-social learning platforms—infrastructure challenges that are often more complex in lower-resource settings. These include the tangible costs associated with hardware, software, and network connections. The intangible costs of the *learning curve* that must be faced by each participant must also be considered in the implementation of mobile-social learning programs as some platforms are inherently more complex and less user-friendly than others.

### Implications for Future Research and Practice

Several key areas of digital health research are needed to support the efficacy of mobile-social learning. The use of digital platforms is well-suited to measuring the utility of the approach given that the platforms allow for the tracking of time and frequency of use by individual learners, including the degree to which these learners take advantage of the opportunities for social interaction as an enhancement of learning. These data are critical for the evaluation of the effectiveness of the mobile-social teaching and learning strategy. In addition, although mobile-social learning offers various advantages and benefits, certain studies in our review (3/31, 10%) did not yield significant learning outcomes among participants, with participants in some studies citing that they preferred face-to-face learning [16,17,49]. Thus, this underscores the importance of providing multifaceted options in CPD to meet the diversity of learning needs and preferences. Although most studies that included virtual mentorship noted positive results and favorability among participants, the health care infrastructure and human resource constraints in many LMICs may limit access and availability of mentors [69]. Thus, further research is needed to assess how mentorship can be provided without being an additional burden on health care systems and personnel. More research is also needed on developing mobile-social learning programs designed to fit local contexts. Future studies should evaluate the use of recent technological innovations such as augmented reality and virtual reality for mobile-social learning. The included studies did not evaluate the use of these platforms; however, these interventions are gaining traction for the training of health care professionals and preclinical students in high-income countries [70]. As technology advances at a rapid pace, there is a need to explore how such technological innovations can be accessible for health care providers and students in lower-resource settings. Although none of our included studies applied a gender lens to the observed outcomes, it is generally acknowledged as a moderating influence in health and education and, thus, should be considered in the implementation of programs [64,71]. More evidence generated through studies that are methodologically rigorous while simultaneously allowing for lean, iterative, and rapid-paced development and evaluation is needed to thoroughly assess the benefits of mobile-social learning in comparison with traditional learning modalities [72].

Central to the success of digital health interventions is the knowledge of health informatics challenges that may be experienced, particularly in environments where instability in the digital and health infrastructure is common [73]. Developing interventions that are designed to meet learning needs and preferences will entail more representation of individuals from LMICs in the technology development sector to inform the development of digital tools that fit local country contexts [74,75]. Although the included studies discussed challenges in internet access as a barrier to accessing mobile-social learning programs, there was limited discussion on the digital environment in which these studies were implemented. When developing, implementing, and expanding the use of digital health programs that focus on mobile-social learning in LMICs, it is essential to foster strong digital health ecosystems by building and promoting partnerships between the relevant public and private sectors [10,76,77]. Communication channels such as WhatsApp are increasingly being used as a simple, low-cost, and effective means of learning and communication within the clinical health sector. However, more attention must be paid to confidentiality, consent, and data security if individual client data are being shared through these channels [78]. The roles and responsibilities of medical professionals when using digital platforms for mobile-social learning must be outlined along with the development of guidelines and protocols to facilitate the integration of mobile-social learning within digital and health care infrastructures [78]. Thus, the standardization of policies in the exchange and use of information between systems will be critical in ensuring the usability and sustainability of mobile-social learning programs [73,78,79].

### Limitations

This study is not without limitations. Although we used a comprehensive search strategy, we cannot guarantee that it identified all relevant studies. However, the incorporation of gray literature in our review reduced publication bias and denoted experiential evidence that supported mobile-social learning. Our search yielded studies that were predominantly focused in sub-Saharan Africa (28/31, 90%), with only a few studies (3/31, 10%) being conducted outside of that global region. Thus, the findings we report cannot be generalized to all LMIC settings given that sociocultural contexts, subjective norms, health system contexts, and digital environments may vary in different country settings. Finally, we only included articles that were published in English; therefore, we could have missed relevant articles published in other languages.

### Conclusions

Mobile-social learning is a particularly useful modality for curriculum dissemination and skill training, and the interface of mobile and social learning offers an interaction effect that can serve as a catalyst for improved learning outcomes coupled with increased social capital. The mobile-social approach is by its nature conducive to the dissemination of shorter segments of key content packed in social platform formats that allow for peer and mentor engagement and interactivity. The concurrent enhancement of mobile curriculum dissemination apps, embedding of proven social interaction strategies into those apps, and development of more and newer user-friendly digital learning opportunities will lead to greater opportunities for learning and peer and mentor support in the interest of improving the quality of health services. As more countries turn to digital modalities of learning, it will be imperative for programs to be adapted for both the technological ecosystem and the local and national health care systems.

## Appendix

Multimedia Appendix 1 Search strategy.

Multimedia Appendix 2 Design and level of evidence of included studies.

Multimedia Appendix 3 Summary of included articles.

## Abbreviations

CPD: continuing professional development
LMIC: low- and middle-income country
mHealth: mobile health
PRISMA: Preferred Reporting Items for Systematic Reviews and Meta-Analyses
