# Supplementary material for: Mobile-Social Learning for Continuing Professional Development in Low- and Middle-Income Countries: Integrative Review
Source: JMIR Med Educ. 2022 Jun 7;8(2):e32614. doi: 10.2196/32614 (PMC9214614; doi:10.2196/32614)
Supplement: Multimedia Appendix 1 [file mededu_v8i2e32614_app1.docx]

Multimedia Appendix 1 : Search strategy

Pubmed:

("Education, Continuing"[Mesh]) OR "Education, Medical, Continuing"[Mesh]) OR "Education, Nursing, Continuing"[Mesh] OR "continuing education" [tiab] OR "e-Learning" [tiab]) AND ("Health Personnel"[Mesh] OR "Physicians"[Mesh] OR "Nurses"[Mesh] OR "Community Health Workers"[Mesh] OR "Healthcare Provider" OR "Nurse*" OR "Midwives" OR "Doctor*" OR "Physician*"[tiab] OR "Nurse" [tiab] OR "Healthcare Provider" [tiab] OR "doctor" [tiab] OR "physician" [tiab])) AND ("Software"[Mesh] OR "Virtual platform" OR "mobile health" OR "Telehealth" OR "Telemedicine" [Mesh] OR "eHealth" OR "mHealth" OR "digital" OR "Virtual training" [tiab] OR "Social Networking"[Mesh] OR "Social Network*" OR "Whatsapp" OR "Facebook" OR "SMS" OR "Text message" [tiab] OR "Whatsapp" [tiab]) AND

"Africa South of the Sahara"[Mesh] OR "sub-Saharan Africa" OR "Developing Countries"[Mesh] OR “Developing country” [tiab] OR “Latin America”[Mesh] OR “Southeast Asia” [Mesh] OR “Caribbean” [Mesh] OR “ low income countries” [tiab] OR “low and middle income countries” [tiab])) AND ("Mentors"[Mesh] OR "Peer groups" OR "Peer learning" OR "mentorship" OR "collaboration" OR "mentor*" [tiab]))

Embase:

('continuing education' OR 'medical education' OR 'nursing education' OR 'e learning' OR 'virtual learning environment') AND (physician OR nurse OR 'health auxiliary' OR midwife OR 'nurse practitioner' OR 'health care personnel') AND ('mobile health' OR mhealth OR telehealth OR telemedicine OR 'digital health' OR 'distance learning' OR 'social media' OR 'text messaging' OR 'mobile phone') AND (africa OR 'africa south of the sahara' OR 'southeast asia' OR 'south and central america' OR caribbean OR 'developing country' OR 'low income country' OR 'middle income country')

CINAHL:

( Health Personnel OR health professional OR nurse OR nurses OR nursing OR physicians OR doctors OR community health workers OR midwives ) AND ( cell phone OR mobile phones OR smart phones OR mobile device OR facebook OR SMS OR Virtual platform OR mobile health OR Telehealth OR Telemedicine OR eHealth OR mHealth OR digital health OR virtual training OR online learning OR education, continuing OR education, medical, continuing OR education, nursing, continuing OR continuing education OR e-Learning ) AND ( developing countries or developing nations or third world or low income countries or least developed countries or ldcs OR middle income countries )

Digital Square:

Digital, mobile learning, mentorship, collaboration , Virtual training, digital training, collaboration, mentorship, social interaction, social media, Whatsapp, Facebook, Twitter, SMS, chat, message groups

USAID mHealth Database:

Virtual training, digital training, collaboration, mentorship, social interaction, social media, Whatsapp, Facebook, Twitter, SMS, chat, message groups
