# Supplementary material for: Mobile-Social Learning for Continuing Professional Development in Low- and Middle-Income Countries: Integrative Review
Source: JMIR Med Educ. 2022 Jun 7;8(2):e32614. doi: 10.2196/32614 (PMC9214614; doi:10.2196/32614)
Supplement: Multimedia Appendix 2 [file mededu_v8i2e32614_app2.docx]

**Appendix 2**

**Table S1. Design and level of evidence of included articles**

| Study Design | Articles | Category ^a^ | Level of Evidence |
| --- | --- | --- | --- |
| Qualitative |  |  |  |
|  | Makwabe et al. [36] | 2 | Level III, Good |
|  | Kabinga- Makukula et al. [30] | 1 | Level III, Good |
|  | Ajuwon et al.[50] | 1 | Level III, Good |
|  | Willemse et al.[32] | 1 | Level III, Good |
|  | Asgary et al. [58] | 1 | Level III, Good |
|  | Asiedu et al. [41] | 1 | Level III, High |
|  | Bertman et al. [8] |  | Level III, Good |
|  | Pimmer et al. [15] | 1 | Level III, Good |
|  | Pimmer et al. [34] | 1 | Level III, Good |
| Quantitative |  |  |  |
|  | Abiodun et al. [37] | 1 | Level III, High |
|  | Pimmer et al.[51] | 1 | Level III, High |
|  | Abawi et al. [1] | 1 | Level III, Low |
|  | Shah et al. [45] | 1 | Level III, Low |
|  | Woods et al.[31] | 1 | Level III, High |
|  | Pollack et al. [54] | 1 | Level III, High |
|  | Hockenberry et al.[46] | 1 | Level III, Low |
|  | Asgary et al. [47] | 1 | Level III, Low |
|  | Allen [48] | 2 | Level V, Good |
|  | Pimmer et al. [51] | 1 | Level II, Good |
|  | Yigzaw et al [16] | 1 | Level II, High |
|  | Biemba et al. [35] | 1 | Level I, High |
| Mixed Methods |  |  |  |
|  | Pimmer et al. [39] | 1 | Level III, Good |
|  | Pimmer et al. [52] | 1 | Level III, Good |
|  | Gross [57] | 2 | Level V, Good |
|  | Peponis et al. [38] | 1 | Level III, Low |
|  | Feldacker et al. [42] | 1 | Level III, Good |
|  | Hoedebecke et al. [43] | 1 | Level III, Low |
|  | Scott et al. [44] | 1 | Level III, Good |
|  | Muhe et al. [49] | 1 | Level II, Good |
|  | Kaphle et al. [53] | 1 | Level I, Good |
|  | Ugwa et al. [17] | 1 | Level I, High |

^a^Category 1= Peer-reviewed studies; Category 2= Organizational case reports, presentations, conference abstract
