# Supplementary material for: Mobile-Social Learning for Continuing Professional Development in Low- and Middle-Income Countries: Integrative Review
Source: JMIR Med Educ. 2022 Jun 7;8(2):e32614. doi: 10.2196/32614 (PMC9214614; doi:10.2196/32614)
Supplement: Multimedia Appendix 3 [file mededu_v8i2e32614_app3.docx]

**Appendix 3**

**Table S2. Summary of included articles**

| Author | Location | Clinical Focus | Participants | Study Aim | Virtual Mentorship | Virtual Peer Engagement | Significant Findings |
| --- | --- | --- | --- | --- | --- | --- | --- |
| **Text message, SMS, MIM interventions (e.g., WhatsApp, Viber)** | | | | | | | |
| Pimmer et al. [15] | Malawi | Combined disciplines (e.g. HIV, nutrition) | CHWs | WhatsApp groups were implemented and researched in Malawi to assess benefits and constraints in MIM use for rural CHWs. | Yes; Group facilitators provided opportunities to share solutions and approaches to clinical cases. | Yes; Participants used WhatsApp to discuss health-related and technical problems along with problem solving. | MIM provided benefits in enhancing communication ease and quality, and increased social capital. |
| Gross [57] | Kenya | Family planning with a focus on healthy timing and spacing of pregnancy | Family planning students and health care providers in Kenya | To evaluate the use of a WhatsApp Messenger group as a learning platform for family planning professional development. | Yes; Group administrators aided in completing training and accessing resources. | Yes; Peer-to-per learning occurred through active discussions on cases | Participants were receptive to using WhatsApp for CPD. increases in knowledge scores were reported. |
| Allen [48] | Multiple countries | Leadership, management, and governance in midwifery | Midwives | To support the implementation of service delivery using integrated peer support networking as a complementary component of a LMG for Midwifery Managers Course. | No | Yes; WhatsApp was used to provide peer support, data exchange, program updates, and service delivery strategies. | WhatsApp networks generated diverse, meaningful, and technically relevant conversations. |
| Muhe et al. [49] | Tanzania | IMCI | Clinicians and nurses | Evaluated results of distance learning IMCI training program in Tanzania as an alternative to the standard training course. | Yes; Mentors interacted with trainees via SMS along with on-site visits. | Yes; Peer learning groups of 2-5 persons was incorporated into the distance learning. | Health care providers trained in distance learning IMCI performed better than participants in the standard course. |
| Ajuwon et al.[50] | Nigeria | General nursing practice | Nursing students | Evaluated feasibility of using WhatsApp to provide supervision and support for student nurse tutors during a teaching practice placement in Nigeria. | Yes; Moderators in WhatsApp groups provided continuous support to students in which students contributions and clinical questions were acknowledged. | Yes; Lively discussions took place among students on WhatsApp with students sharing experiences and creating opportunities to ask questions. | WhatsApp-enabled learning spaces were valuable for the transfer and application of knowledge in day-to-day teaching and practice. |
| Pimmer et al. [51] | Nigeria | General nursing clinical knowledge | Nursing students | Examined the use of WhatsApp by nursing students during placements and potential associations with socio-professional indicators. | Yes; Group facilitators developed a guide of clinical topics to discuss with students and engaged in group discussions. | Yes; WhatsApp was used for peer-to-peer interaction in case discussions, increasing knowledge, and increasing social capital. | WhatsApp strongly enhanced communication with peers and mentors. WhatsApp was positively associated with maintained social capital. |
| Pimmer et al. [52] | Multiple countries | Combined disciplines including maternal health | CHWs, nurses, midwives, students, physicians, lab tech, researchers, policymakers | Examined the ways in which MIM platforms are being used for learning and knowledge sharing and identify associated potentials and constraints. | Yes; WhatsApp groups were used for supervision and team management. | Yes; Peer to peer engagement and learning through MIM | MIM spaces facilitated opportunities for knowledge creation and sharing, supervision, problem solving, and enacting social presence for students that fostered collaboration. |
| Bertman et al. [8] | Zimbabwe | Adolescent HIV/AIDS | Nurses and counselors | Assessed the text-message component of a blended training program in HIV counseling and testing, with a focus on active learning and teamwork using minimal resources. | Yes; Mentors provided feedback and suggestions on participant cases on WhatsApp in addition to sending encouragement and reminders to participants. | Yes; Social interactions, check-ins, real-time usage on WhatsApp to discuss workplace issues, clinical cases, questions, advice, and support. | Participants strongly endorsed using WhatsApp groups as part of the training. The groups facilitated peer support, learning, and problem solving. |
| Kabinga-Makukula et al.[30] | Zambia | Sexual and reproductive health and primary care | Nurses and midwives | Explored the use of instant messaging in continuing education for rural nurse managers. | No | Yes; WhatsApp used to build team-based relationships, offer clinical support, and professional networking. | WhatsApp enhanced communication, promoted social interaction, and supported the application of knowledge to practice. |
| Woods et al.[31] | South Africa | HIV/AIDS, TB, Maternity/PMCT | Physicians | Evaluated clinicians' use of a WhatsApp chat group as a learning tool, assessed clinicians' confidence in managing complicated HIV and TB patients, described the perceived usefulness of the chat group as a learning tool, and evaluated clinicians' knowledge and use of informed consent when sharing case details on WhatsApp. | No | Yes; WhatsApp facilitated group engagement and peer case discussions. | Most participants gained new clinical confidence from group participation. Students who regularly engaged in the group were able to extend their current knowledge base. |
| Willemse et al.[32] | South Africa | General nursing practice including primary care and clinical skills | Nursing students | Explored the experiences of nursing students who participated in an authentic mobile learning enactment aimed at enhancing their learning experiences. | No | Yes; WhatsApp chat groups with peers  supported collaborative learning. | Mobile devices afforded a learning platform that enhanced engagement and allowed for flexibility in time allocated to complete tasks and learning. |
| Pimmer et al.[33] | Nigeria | General nursing clinical knowledge | Graduate nursing students | Examined the use of WhatsApp in aiding new graduate nurses transition into their clinical roles and with professional development. | Yes; Moderators initiated topics of discussion and acknowledged and responses to participants contributions. | Yes; Peers interacted with another to facilitate knowledge transfer and increase social capital and peer-support. | Participants in WhatsApp groups had significantly higher knowledge levels and fewer feelings of professional isolation. |
| Pimmer et al. [34] | South Africa | General nursing clinical knowledge | Graduate nursing students | Assessed MIM to engage young professionals in mobile-learning communities during their school-to-work transition. | Yes; Moderators constructed environments to facilitate interaction and learning within the WhatsApp groups | Yes; WhatsApp groups enabled participants to be active in cooperative problem solving along with providing socio-emotional support. | Participants stated the ability to share information and knowledge was associated with learning and the development of professional knowledge. |
| Biemba et al.[35] | Zambia | Integrated community case management of pneumonia, diarrhea, and malaria | CHWs | Assessed the use of mobile technology in addressing challenges to successful ICCM implementation. | Yes; Supervisors provided feedback to CHWs on referral case outcomes via SMS. | No | The intervention was associated with 18.0% improvement in supportive mentor supervision and 21.0% increase in the appropriate treatment for pneumonia, however these changes were not statistically significant. |
| Makwabe et al. [36] | Rwanda and Tanzania` | Nephrology; hemodialysis | Nurses, physicians | Content within a WhatsApp group developed for hemodialysis nurses was audited to determine topics of interest and use for learning. | No | Yes; WhatsApp chat groups fostered clinical case discussions, educational videos, educational instructions, textbooks and notes. | High uptake of practical case discussions were reported on WhatsApp. |
| Abiodun et al.[37] | South Africa | General nursing practice | Nursing students recently graduated from a BSN program | WhatsApp was investigated to evaluate its impact in supporting graduate nurse student in transitioning into nursing practice. | No | Yes; The WhatsApp group supported nurse graduates in developing social capital and reducing social isolation | Mutual learning and awareness, and improved social capital were noted. |
| Peponis et al. [38] | Ethiopia | Orthopedics | Resident physicians | Evaluated a modern, engaging, and easily accessible approach to medical education through a virtual journal club to physician trainees through instant messaging on Viber. | Yes; Senior members posed quiz questions with feedback. | Yes; Viber fostered communication between peers with a highlight on case discussions. | Research skills and clinical practice improved. 95% of participants preferred mobile-social learning compared to traditional learning. |
| **Social media (e.g. Facebook)** | | | | | | | |
| Pimmer et al. [39] | South Africa | Midwifery research skills | Nurses | Investigated the use of a Facebook group as a distance learning tool over time. | Yes; Mentors supervised participants on Facebook and provided feedback, communicated goals, and posted material relevant to research concepts and training. | Yes; Participants interacted with peers via Facebook groups. | Social media for learning was well received. Participants highlighted the educational value in Facebook for facilitating knowledge and sharing ideas with instructors and peers. |
| **Text Messaging with Phone Calls** | | | | | | | |
| Asgary et al.[40] | Ghana | Cervical cancer screening | Nurses | Explored acceptability and feasibility of smartphone-based training of low-level to mid-level health professionals in cervical cancer screening using visual inspection with acetic acid (VIA)/cervicography. | Yes; Mentorship occurred through text messages in which participants sent cervical imaging to mentors who provided feedback. | No; However, participants suggested peer support network to discuss pictures and cases | Smartphone-based training and mentorship was perceived as an important and essential complementary process to further develop diagnostic and management competencies for participants. |
| Asiedu et al.[41] | Ghana | BEmONC | Doctors, midwives | Explored health care worker’s experiences of mobile mentoring and training gin improving newborn survival rates in Ghana. | Yes; Master mentors conducted mentoring through calls and SMS messages in addition to facilitating onsite training. | No | Mentors contributed to a conductive learning environment conducive to learning, and provided opportunity for professional growth and quality improvement. |
| Yigzaw et al. [16] | Ethiopia | EmONC | Midwife, nurse, health officer | This study evaluated whether a blended learning approach using daily SMS and weekly phone calls to in-service EmONC training could be as effective as a conventional learning approach while reducing costs. | Yes; Mentoring occurred through phone calls at least once a week, during which trainers discussed difficult cases encountered in the real clinic setting. | No | Knowledge scores were similar for the blended and conventional learning groups at three months post-training (74.7% vs 75.5% = 0.720). Post-training skills scores were significantly higher for the conventional than the blended learning group (85.8% vs 75.3%, *P* < 0.001). |
| Ugwa et al. [17] | Nigeria | BEmONC | CHWs, physicians, nurses, other | Compared health workers knowledge and skills competencies between blended learning and traditional learning. | Yes; Mentorship occurred through text messages and phone calls. | No; Peer interaction only occurred onsite and was not virtual. | The acquisition and retention of BeMONC skills were improved amongst participants in the mobile-mentoring arm. Mobile mentoring contributed to clinical confidence. |
| **Phone Calls** | | | | | | | |
| Kaphle et al. [53] | India | Malnutrition | CHWs | Evaluated improving CHW performance through providing individual feedback to CHWs using an mHealth program to address child malnutrition. | Yes; Community nutrition experts conducted phone calls with participants to discuss feedback along with performance indicators. | No | A moderate to large effect was found in performance feedback resulting in participants having improved motivation and performance. |
| **MOOC platforms with discussion forums, social media, video, and/or mobile messaging** | | | | | | | |
| Abawi et al. [1] | Multiple countries | Sexual and reproductive health | Nurse, midwifes, physicians, public health specialists, other | Assessed learning outcomes of an online course that used social media (Google groups, Facebook, Twitter) to facilitate learning and mentorship. | Yes; Personal coach/tutor was assigned to each learner with the aim of adapting course to needs of students | Yes; Interaction between students on social platforms using Google Groups, Facebook, and Twitter. | The course positively impacted participants’ knowledge. Ongoing engagement with course coaches was well-received. |
| Feldacker et al. [42] | Multiple countries | HIV/AIDS | Physicians, nurses, clinical/medical officer, nursing assistant, other | Evaluated student experiences using an online Clinical Management of HIV course that facilitated online commentary and knowledge exchange between students. | No; Mentoring was not provided however participants suggested including mentoring opportunities. | Yes; Interactive online discussions and commentary with peers. | Participants noted knowledge gains, the flexibility of the format, along with highlighting the interactive modalities of learning. |
| Hoedebecke et al. [43] | Multiple countries | Primary Care | Physicians | Evaluated the combination of a MOOC with social media to facilitate interaction between students and improve completion rates. | Yes; Mentoring through WhatsApp and Facebook groups contributed to improved knowledge outcomes. | Yes; Facebook and WhatsApp augmented learning through increased discussion on a global scale. | Improved course completion rates, the ability to collaborate, unlimited course accessibility, and resultant knowledge gains were benefits of the program. Course completion rates were five times greater than standard MOOCs. |
| Scott et al.[44] | Rwanda | Not specified | Health care providers, , hospital administrators, researchers, data analysts | Assessed learning outcomes from a blended learning course that was coupled with interaction through social media (e.g. Twitter) and discussion boards. | Yes; Live twitter sessions occurred with course faculty in which participants were able to obtain feedback. | Yes; Peer interaction occurred on discussion boards. | The program had high rates of completion with the majority (81%) strongly agreeing that the program helped them in improving quality of care. |
| Shah et al. [45] | Nepal | Anesthesia | Non-Physicians | This study used distance learning coupled with structured support through SMS and phone calls to train students on anesthesia. | Yes; Mentors communicated with participants regularly through text messaging and phone calls to reinforce content and continued education throughout the program. | No | The program increased student motivation while promoting strong educational mentorship. |
| Hockenberry et al.[46] | Multiple countries | Pediatric hematology-oncology | Nurses | Learning outcomes of an online course coupled with Zoom and WhatsApp sessions to reinforce independent learning experiences were evaluated. | Yes; Teaching sessions on Zoom and WhatsApp reinforced independent learning experiences. Nurses received feedback from educators throughout the program regarding their progress and developing independent nursing skills. | Yes; Peer interaction occurred through questions posted on discussion forums along with Zoom and WhatsApp interactions. | Participants reported substantial knowledge gains throughout the program. |
| **Telemedicine Platforms with Videoconferencing** | | | | | | | |
| Asgary et al. [47] | Eswatini | Cervical cancer screening | Nurses | Evaluated the impact of smartphone-based strategies including mentorship in improving reliability, reproducibility, and quality of VIA in humanitarian settings. | Yes; Mentorship provided to participants through feedback on smartphone cervical imaging. | No | Smartphone mentorship provided experiential learning to improve competencies. |
| Pollack et al. [54] | Vietnam | HIV/AIDS | Physicians | A telehealth program that fostered didactic training, case-based peer-to-peer learning, and distance mentoring from HIV experts was assessed for health care workers in rural areas of Vietnam. | Yes; Mentors provided short didactic presentations on topics chosen based on participants' needs. | Yes; Peer learning occurred through webinars, discussions, and case-based learning. | Over 85% of respondents agreed that access to the telehealth program improved the quality of care they provided to their patients. |

BEmONC : Basic emergency obstetric and newborn care

CHW: Community health workers

EmONC: Emergency obstetric and newborn care

IMCI : Integrated management of childhood illness

PMCT : Prevention of mother-to-child transmission

MIM: Mobile instant messaging

SMS: Short message services
